# Association between nutritional status and sepsis-induced coagulopathy: a multicenter retrospective cohort study

**DOI:** 10.3389/fnut.2026.1894405

**Published:** 2026-08-10

**Authors:** Jiyuan Zhang, Lujia Tang, Zhihui Feng, Chaoping Ma, Peixian Xu, Jiawei Ye, Chengjin Gao

**Affiliations:** The Department of Emergency, Xinhua Hospital, Shanghai Jiao Tong University School of Medicine, Shanghai, China

**Keywords:** eICU database, GNRI, MIMIC-IV database, PNI, sepsis-induced coagulopathy

## Abstract

**Background:**

Malnutrition and sepsis-induced coagulopathy (SIC) are common and critical complications in septic patients. This study aimed to evaluate the association between nutritional status and SIC, as well as their combined effects on clinical outcomes in adult septic patients.

**Methods:**

This retrospective study extracted data from the MIMIC-IV v3.1 and eICU databases, and additionally enrolled adult patients with sepsis who received treatment at Xinhua Hospital Affiliated to Shanghai Jiao Tong University School of Medicine from 2021 to October 2022. Baseline clinical characteristics were compared between patients with and without SIC, and between patients with and without nutritional risk. Logistic regression models were constructed to identify independent risk factors for SIC, and restricted cubic spline curves were plotted to visualize dose–response relationships. The Random Forest machine learning method was used to evaluate the association between nutritional indicators and SIC. We used Spearman correlation analysis to investigate the relationship between nutritional indicators and SOFA and SIC scores, as well as coagulation indicators. The associations among nutritional indicators were assessed using logistic proportional hazards models, and interactions were examined on both additive and multiplicative scales.

**Results:**

Patients with SIC had lower Geriatric Nutritional Risk Index (GNRI) and Prognostic Nutritional Index (PNI) scores and higher mortality rates, with statistically significant differences observed in the MIMIC and eICU cohorts (*p* < 0.001). Concurrent low GNRI and PNI levels correlated with higher SIC risk. In the eICU cohort, univariate and multivariate regression analyses confirmed that dual low nutritional indices independently increased SIC risk (OR = 2.24, 95% CI: 1.76–2.86, *p* < 0.001; adjusted OR = 2.18, 95% CI: 1.67–2.85, *p* < 0.001). Restricted cubic splines suggested that patients with higher GNRI and PNI scores have a lower probability of SIC occurrence. The MIMIC database also showed similar results. Spearman analysis revealed positive correlations among the nutritional indicators, as well as negative correlations with the SOFA and SIC scores. No significant multiplicative or additive interactions were identified between GNRI and PNI.

**Conclusion:**

Malnutrition and SIC are associated with higher mortality rates in sepsis. Nutritional status is independently associated with the risk of SIC in adults.

## 1 Introduction

Sepsis is defined as life-threatening organ dysfunction due to a dysregulated host reaction to infection. Sepsis has long been a hot issue in global public health owing to its high mortality rate (1, 2). Approximately 70% of cases of sepsis lead to coagulopathy (3, 4), and among these patients, about 35% may develop disseminated intravascular coagulation (DIC), which exacerbates organ failure and increases mortality (5, 6). Early detection and appropriate management of DIC contribute greatly to improving the clinical outcomes of septic patients (7, 8).

The pathogenesis of sepsis-induced coagulopathy (SIC) is primarily associated with activation of the coagulation pathway, impairment of the anticoagulant system, inhibition of fibrinolysis, and platelet aggregation in patients with sepsis (9, 10). SIC represents an early stage of DIC; in patients with early-stage coagulation dysfunction, the coagulation state remains reversible (11). The 2017 SIC score proposed by the International Society on Thrombosis and Haemostasis (ISTH) was specifically designed to identify sepsis patients in the early stages of DIC and improve patient outcomes (12). Therefore, early identification of patients at risk of developing SIC is crucial for improving outcomes and facilitating the development of more personalized management and treatment strategies.

Patients with malnutrition may are prone to a higher incidence of complications, longer hospital stays, and poorer outcomes (13–15). Consequently, numerous indicators have been developed to assess nutritional status. However, these methods require subjective judgment and the collection of complex data, and may not provide a timely assessment of the nutritional status of critically ill patients (16, 17). In contrast, several simple and readily accessible nutritional assessment tools have been widely applied in clinical practice. The Geriatric Nutritional Risk Index (GNRI) assesses the risk of malnutrition based on serum albumin levels, height, and weight parameters; the Prognostic Nutritional Index (PNI) evaluates nutritional status using serum albumin concentration and peripheral blood lymphocyte count. Previous studies have demonstrated that these nutritional markers can predict the prognosis of conditions such as sepsis, cancer, and cardiovascular disease (18–20). Nevertheless, although the nutritional status of patients with sepsis is routinely assessed, previous studies have not comprehensively explored the correlation between nutritional status and SIC in septic patients.

We utilized large-scale, multicenter intensive care unit databases—specifically the eICU Collaborative Research Database (eICU-CRD) (21) and the Medical Information Mart for Intensive Care IV (MIMIC-IV) (22)—and combined them with data on sepsis patients admitted to the Department of Emergency Medicine at Xinhua Hospital, affiliated with Shanghai Jiao Tong University School of Medicine, between 2021 and October 2022 to conduct a comprehensive retrospective analysis. Considering that patients with malnutrition tend to have more severe disease and potentially poorer prognoses, this study aimed to evaluate the correlation between nutritional status and SIC, as well as their impact on outcomes in adult patients with sepsis, in order to provide evidence for targeted nutritional supplementation strategies in future research.

## 2 Method

### 2.1 Data sources

We retrospectively enrolled septic patients who were admitted to the emergency department at Xinhua Hospital, Affiliated to Shanghai Jiao Tong University School of Medicine, from 2021 through October 2022. The study design was approved by the Ethics Committee at Xinhua Hospital (No. XHEC-D-2026-65) with informed consent being waived as the research involved no more than minimal risk to participants. The study complied with the ethical guidelines of the Declaration of Helsinki. This retrospective study also utilized two intensive care unit databases: the Medical Information Mart for Intensive Care version IV 3.1 (MIMIC-IV v3.1) and the eICU Collaborative Research Database version 2.0 (eICU-CRD 2.0). The MIMIC-IV database contains electronic health records of more than 90,000 adult ICU patients hospitalized at Beth Israel Deaconess Medical Center in Boston between 2008 and 2022. Developed by the Philips eICU Research Institute, eICU-CRD 2.0 is a multicenter database that includes clinical data from more than 200 hospitals throughout the United States collected between 2014 and 2015. All data within both databases were fully de-identified; therefore, formal informed consent from individual patients was not required for analysis.

### 2.2 Study population

We included adult patients aged ≥18 years with a first ICU admission and a confirmed diagnosis of sepsis. Sepsis was diagnosed in accordance with the Sepsis-3.0 criteria (1). SIC Diagnostic Criteria: According to the ISTH-SIC diagnostic criteria (12), a score of four or higher is diagnostic of SIC. Patients were excluded if their ICU length of stay was shorter than 24 h or if key nutritional and coagulation laboratory data were incomplete.

### 2.3 Data collection

We used the structured query language (SQL) of Navicat premium 17.1.0 to collect data from the MIMIC-IV database and eICU database. The following variables were extracted for each patient: (1) demographics, including age, sex, height, and weight; (2) comorbidities, including diabetes, kidney disease, and severe liver disease; (3) laboratory tests on the day of ICU admission, including lymphocyte count, hemoglobin (Hb), platelets (Plt), white blood cell (WBC) count, red blood cell distribution Width (RDW), mean corpuscular volume (MCV), albumin (Alb), serum creatinine (Scr), international normalized ratio (INR), total bilirubin (TBIL), alanine aminotransferase (ALT) and other indicators; (4) SOFA scores, SIC scores, GNRI and PNI scores; (5) clinical outcomes, including length of hospital stay, length of stay in the ICU, 28-day mortality, and the incidence of SIC. The above indicators were from the patient's first test after admission to the ICU.

GNRI was calculated as: GNRI = [14.89 × serum albumin (g/dL)] + [41.7 × (actual BMI/ideal BMI)] (23, 24), with the ideal BMI defined as 22 kg/m^2^(24). According to the GNRI, nutritional risk can be classified as follows: No nutritional risk (GNRI ≥98), Low risk (92 ≤ GNRI < 98), Moderate risk (82 ≤ GNRI < 92), and High risk (GNRI < 82). PNI was calculated as: serum albumin (g/L) + 5 × lymphocyte count (10^9^/L) (25). According to the PNI, nutritional risk can be classified as follows: No nutritional risk (PNI > 38), Moderate risk (35 ≤ PNI ≤ 38) and High risk (PNI < 35).

### 2.4 Statistical analysis

There were no missing values for baseline albumin, lymphocyte count, nutritional indices, platelet count, or INR in any of the patients included in the study. Multiple imputation *via* chained equations with predictive mean matching was performed, generating five complete independent datasets to handle remaining missing variables. Variables with a missing rate exceeding 20% were excluded from all analyses.

Continuous variables were expressed as median (interquartile range, IQR), while categorical variables were summarized as count (percentage). Between-group differences were analyzed using the Mann–Whitney U-test for continuous variables. Categorical variables were compared with the χ^2^ test or Fisher's exact test where applicable, with results presented as proportions. We used univariate and multivariable logistic regression analyses to assess the relationship between nutritional indicators and the incidence of SIC. We fitted restricted cubic splines to evaluate the dose-response relationship between GNRI/PNI and SIC risk. We used the Random Forest machine learning method to evaluate the relationship between nutritional indicators and SIC, ranked by importance. We examined the interactions among nutritional indicators both at the additive interaction level [by calculating the relative excess risk of interaction (RERI) and the attributable proportion (AP)] and at the multiplicative interaction level (by including product terms). Finally, we used Spearman correlation analysis to examine the correlations between nutritional indicators and the components of the SIC scores.

We set the eICU database as the primary cohort and the other two databases as validation cohorts. All statistical tests were two-tailed, and statistical significance was defined at α < 0.05. All statistical analyses were conducted in R software (version 4.4.1).

## 3 Results

### 3.1 Patient eligibility outline and baseline characteristics

**Figure 1:**
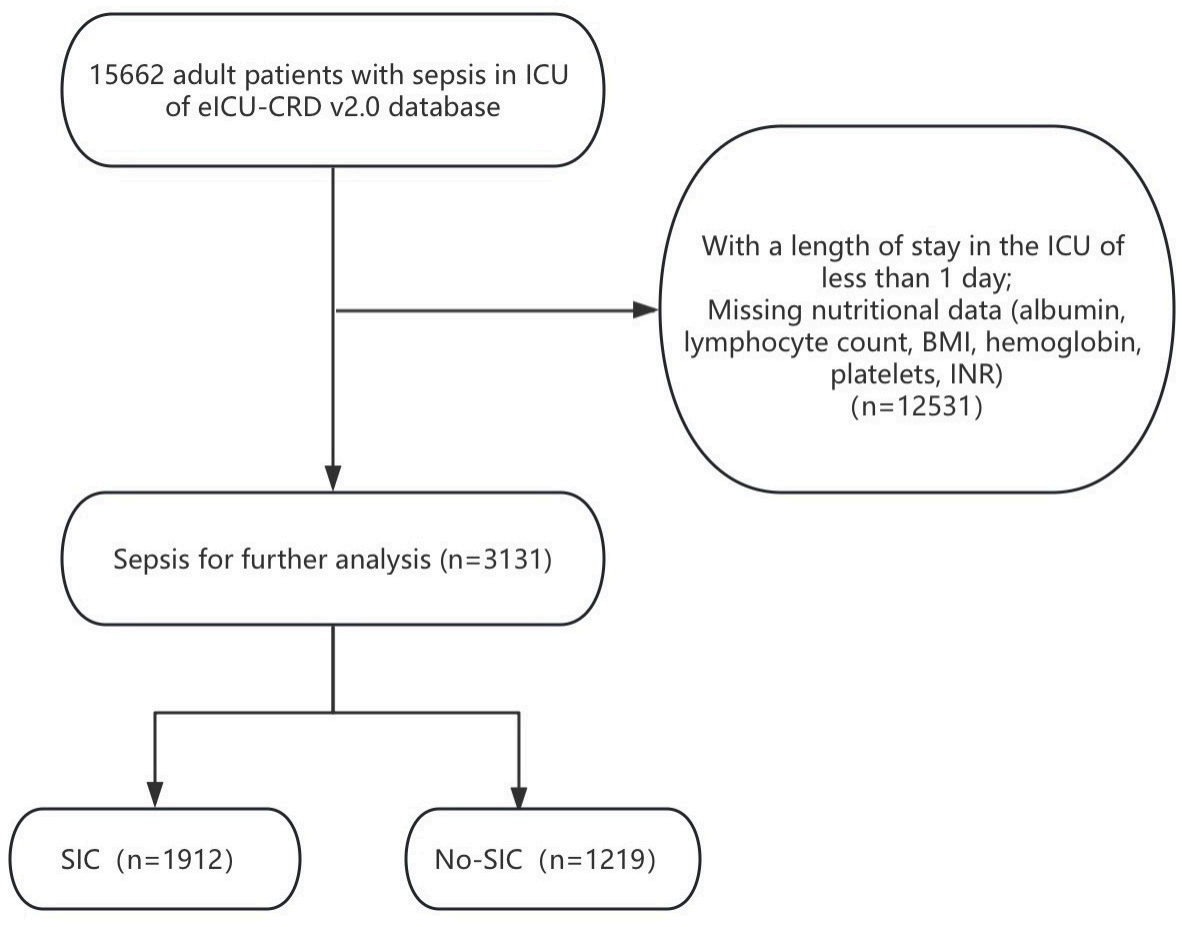
eICU-CRD database screening flowchart.

A total of 15,662 adult patients with sepsis from the eICU database and 28,235 adult patients with sepsis from the MIMIC database were initially screened for this study. According to the inclusion criteria, a total of 4,811 patients with sepsis were included in the analysis, comprising 3,131 from the eICU-CRD, 1,466 from the MIMIC-IV database and 214 from Xinhua Hospital, Affiliated to Shanghai Jiao Tong University School of Medicine. Figure 1 illustrates the screening process for the eICU database, while Figure 1 in the Supplementary materials shows the flowchart for the MIMIC database. Table 1 presents a baseline comparison of data from the eICU database, grouped by the presence or absence of SIC. Patients in the SIC group exhibited significantly higher SOFA scores (median 8 vs. 6, *p* < 0.001) and 28-day mortality rates (23.95 vs. 15.34%, *p* < 0.001), a higher prevalence of pre-existing liver and kidney disease, and lower GNRI (median 90.36 vs. 93.13, *p* < 0.001) and PNI scores (median 28.89 vs. 31.62, *p* < 0.001). Supplementary Tables 1 and 2 summarized the baseline information for the MIMIC and Xinhua Hospital cohorts, respectively. With the exception of the history of kidney disease, the MIMIC database yielded the same conclusions as the eICU database. In the Xinhua Hospital cohort, patients with SIC showed lower GNRI (median: 88.96 vs. 93.30, *p* = 0.401) and PNI (median: 33.88 vs. 35.67, *p* = 0.100) scores, though the differences failed to reach statistical significance. We considered GNRI greater than 98 and PNI greater than 38 to indicate no nutritional risk. Based on this, we divided the participants into four groups: those with both high GNRI and PNI, those with both low GNRI and PNI, those with a high GNRI and low PNI, and those with a low GNRI and high PNI. Table 2 summarized baseline information from the eICU database organized according to the above groupings. The dual low GNRI/PNI group had higher SOFA (median 8 vs. 7, *p* = 0.019) and SIC scores (median 4 vs. 3, *p* < 0.001), a higher 28-day mortality rate (21.99 vs. 15.52%, *p* = 0.023), and a higher incidence of SIC (64.28 vs. 44.52%, *p* < 0.001) compared to the high nutritional index group. Supplementary Tables 3 and 4 displayed the baseline information from the MIMIC and Xinhua Hospital cohorts, stratified into the four groups described above. Consistent trends were observed in both cohorts, despite some non-significant differences.

**Table 1:** Baseline information in the eICU database, grouped by the occurrence of SIC.

Variables	Total (*n* = 3,131)	No-SIC (*n* = 1,219)	SIC (*n* = 1,912)	*P*
Age (years)	66.00 (55.00, 77.00)	65.00 (55.00, 77.50)	67.00 (56.00, 77.00)	0.486
Gender, *n*(%)				0.858
Female	1,409 (45.00)	551 (45.20)	858 (44.87)	
Male	1,722 (55.00)	668 (54.80)	1,054 (55.13)	
Height (cm)	170.00 (162.60, 177.80)	168.00 (162.60, 177.80)	170.00 (162.50, 177.80)	0.555
Weight (kg)	80.28 (65.40, 98.00)	79.70 (65.20, 98.05)	80.70 (65.57, 98.00)	0.751
Body mass index (kg/m^2^)	27.66 (23.30, 33.67)	27.48 (22.96, 33.82)	27.68 (23.54, 33.60)	0.786
Heart rate (bpm)	98.00 (86.00, 108.00)	98.00 (86.00, 108.00)	98.00 (87.00, 109.00)	0.148
Lymphocyte count ( × 10^9^/*L*)	0.81 (0.47, 1.32)	0.90 (0.54, 1.41)	0.76 (0.43, 1.26)	< 0.001
Hb (g/dL)	10.30 (8.70, 11.90)	10.70 (9.10, 12.30)	10.00 (8.60, 11.60)	< 0.001
Plt ( × 10^9^/*L*)	173.00 (113.00, 247.00)	219.00 (171.00, 288.00)	134.00 (83.00, 207.00)	< 0.001
WBC ( × 10^9^/*L*)	13.40 (8.60, 19.40)	14.31 (9.71, 19.96)	12.70 (7.80, 19.02)	< 0.001
RDW (%)	15.80 (14.40, 17.10)	15.30 (14.10, 16.40)	16.00 (14.70, 17.60)	< 0.001
MCV (fL)	91.00 (86.90, 95.30)	91.00 (87.00, 95.00)	91.00 (86.80, 95.90)	0.357
Alb (g/dL)	2.50 (2.10, 3.00)	2.60 (2.20, 3.10)	2.40 (2.00, 2.90)	< 0.001
AST (U/L)	48.00 (25.00, 143.00)	38.00 (22.00, 90.00)	57.00 (29.00, 193.25)	< 0.001
TBIL (mg/dL)	0.90 (0.50, 1.70)	0.70 (0.40, 1.27)	1.10 (0.60, 2.10)	< 0.001
INR	1.40 (1.20, 1.90)	1.20 (1.10, 1.30)	1.70 (1.50, 2.50)	< 0.001
Scr (mg/dL)	1.59 (0.98, 2.72)	1.37 (0.88, 2.46)	1.77 (1.04, 2.90)	< 0.001
Lac (mmol/L)	2.80 (1.50, 2.90)	2.30 (1.20, 2.90)	2.90 (1.70, 3.20)	< 0.001
SOFA score	7.00 (5.00, 10.00)	6.00 (4.00, 9.00)	8.00 (5.75, 11.00)	< 0.001
GNRI	91.36 (79.28, 104.58)	93.13 (81.30, 107.41)	90.36 (78.32, 102.93)	< 0.001
PNI	29.99 (25.02, 35.10)	31.62 (26.79, 36.84)	28.89 (24.07, 33.75)	< 0.001
History of liver disease, *n*(%)				< 0.001
No	2,865 (91.50)	1,172 (96.14)	1,693 (88.55)	
Yes	266 (8.50)	47 (3.86)	219 (11.45)	
History of kidney disease, *n*(%)				0.042
No	2,472 (78.95)	985 (80.80)	1,487 (77.77)	
Yes	659 (21.05)	234 (19.20)	425 (22.23)	
Length of hospital stay (days)	8.00 (4.00, 14.00)	8.00 (5.00, 14.00)	8.00 (4.00, 14.00)	0.078
Length of ICU stay (days)	3.00 (2.00, 6.00)	3.00 (2.00, 7.00)	3.00 (2.00, 6.00)	0.152
Outcome 28d, *n*(%)				< 0.001
Alive	2,486 (79.40)	1,032 (84.66)	1,454 (76.05)	
Death	645 (20.60)	187 (15.34)	458 (23.95)	

Hb, hemoglobin; Plt, platelets; WBC, white blood cell; RDW, red blood cell distribution Width; MCV, mean corpuscular volume; Alb, albumin; AST, aspartate aminotransferase; TBIL, total bilirubin; INR, international normalization ratio; Scr, serum creatinine; Lac, lactate; SOFA, sequential organ failure assessment; GNRI, geriatric nutritional risk index; PNI, prognostic nutritional index.

**Table 2:** Baseline information in the eICU database, grouped by the occurrence of nutritional risk.

Variables	Both high-group (*n* = 310)	High GNRI (*n* = 813)	High PNI (*n* = 166)	Both low-group (*n* = 1,842)	*P*
Age (years)	63.00 (53.00, 74.00)	64.00 (55.00, 74.00)	66.00 (53.00, 82.00)	68.00 (57.00, 79.00)	< 0.001
Gender, *n*(%)					0.007
Female	132 (42.58)	405 (49.82)	80 (48.19)	792 (43.00)	
Male	178 (57.42)	408 (50.18)	86 (51.81)	1,050 (57.00)	
Height (cm)	170.20 (162.60, 177.95)	167.60 (162.00, 177.80)	167.62 (162.50, 177.60)	170.00 (162.60, 177.80)	0.366
Weight (kg)	94.40 (82.23, 110.15)	107.00 (92.80, 123.30)	65.30 (54.40, 77.40)	71.20 (60.50, 82.57)	< 0.001
Body mass index (kg/m^2^)	32.35 (28.59,37.48)	37.42 (33.20,42.78)	22.80 (20.53,25.88)	24.88 (21.86,27.96)	< 0.001
Heart rate (bpm)	98.00 (87.00, 105.75)	98.00 (84.00, 108.00)	98.00 (91.00, 107.75)	98.00 (87.00, 108.00)	0.277
Lymphocyte count ( × 10^9^/*L*)	1.41 (0.93, 2.20)	0.77 (0.49, 1.14)	2.10 (1.38, 3.61)	0.71 (0.41, 1.13)	< 0.001
Hb (g/dL)	11.70 (9.83, 13.28)	10.70 (9.10, 12.20)	10.50 (9.00, 12.10)	9.90 (8.60, 11.50)	< 0.001
Plt ( × 10^9^/*L*)	197.00 (145.00, 260.50)	171.00 (117.00, 239.00)	193.00 (129.75, 257.75)	167.00 (103.00, 246.00)	< 0.001
WBC ( × 10^9^/*L*)	15.10 (10.72, 22.45)	13.30 (8.60, 18.40)	15.40 (11.03, 21.60)	13.10 (8.00, 19.08)	< 0.001
RDW (%)	14.90 (13.90, 16.40)	15.90 (14.50, 17.20)	15.50 (14.22, 16.98)	16.00 (14.60, 17.20)	< 0.001
MCV (fL)	91.10 (87.30, 95.07)	91.00 (86.90, 95.00)	91.95 (86.90, 96.00)	91.00 (86.80, 95.47)	0.903
Alb (g/dL)	3.50 (3.20, 3.70)	2.70 (2.40, 3.00)	3.10 (2.70, 3.40)	2.30 (1.90, 2.60)	< 0.001
AST (U/L)	48.50 (25.00, 175.50)	49.00 (25.00, 166.00)	40.00 (22.00, 100.50)	48.00 (26.00, 139.00)	0.179
TBIL (mg/dL)	0.80 (0.50, 1.70)	1.00 (0.50, 1.70)	0.60 (0.43, 1.20)	0.90 (0.50, 1.70)	< 0.001
INR	1.30 (1.10, 1.70)	1.41 (1.20, 1.93)	1.30 (1.19, 1.90)	1.44 (1.20, 1.91)	< 0.001
Scr (mg/dL)	1.50 (0.96, 2.81)	1.80 (1.10, 2.99)	1.31 (0.90, 2.59)	1.52 (0.92, 2.63)	< 0.001
Lac (mmol/L)	2.70 (1.40, 2.90)	2.50 (1.40, 2.90)	2.90 (1.60, 3.38)	2.90 (1.60, 2.90)	0.002
SOFA score	7.00 (4.00, 10.00)	8.00 (5.00, 11.00)	7.00 (5.00, 9.00)	8.00 (5.00, 10.00)	0.019
PNI	42.18 (39.99, 45.67)	31.52 (27.96, 34.48)	40.92 (39.32, 43.96)	27.05 (22.66, 31.07)	< 0.001
GNRI	112.45 (104.69, 123.18)	108.97 (102.73, 120.29)	89.53 (83.53, 94.40)	82.31 (73.53, 89.98)	< 0.001
SIC score	3.00 (2.00, 4.00)	4.00 (3.00, 5.00)	4.00 (2.00, 4.00)	4.00 (3.00, 5.00)	< 0.001
History of liver disease, *n*(%)					0.009
No	293 (94.52)	758 (93.23)	154 (92.77)	1,660 (90.12)	
Yes	17 (5.48)	55 (6.77)	12 (7.23)	182 (9.88)	
History of kidney disease, *n*(%)					0.030
No	249 (80.32)	612 (75.28)	133 (80.12)	1,478 (80.24)	
Yes	61 (19.68)	201 (24.72)	33 (19.88)	364 (19.76)	
Length of hospital stay (days)	7.00 (4.00,14.00)	8.00 (5.00,14.00)	7.00 (4.00,11.00)	8.00 (4.00,14.00)	0.028
Length of ICU stay (days)	3.00 (2.00,6.00)	3.00 (2.00,6.00)	2.00 (1.00,6.00)	3.00 (2.00,6.00)	0.195
Outcome28d, *n*(%)					0.023
Alive	265 (85.48)	652 (80.20)	132 (79.52)	1,437 (78.01)	
Death	45 (14.52)	161 (19.80)	34 (20.48)	405 (21.99)	
SIC, *n*(%)					< 0.001
No	172 (55.48)	309 (38.01)	80 (48.19)	658 (35.72)	
Yes	138 (44.52)	504 (61.99)	86 (51.81)	1,184 (64.28)	

Hb, hemoglobin; Plt, platelets; WBC, white blood cell; RDW, red blood cell distribution Width; MCV, mean corpuscular volume; Alb, albumin; AST, aspartate aminotransferase; TBIL, total bilirubin; INR, international normalization ratio; Scr, serum creatinine; Lac, lactate; SOFA, sequential organ failure assessment; GNRI, geriatric nutritional risk index; PNI, prognostic nutritional index; SIC, sepsis-induced coagulopathy.

### 3.2 Association between nutritional status and SIC

**Figure 2:**
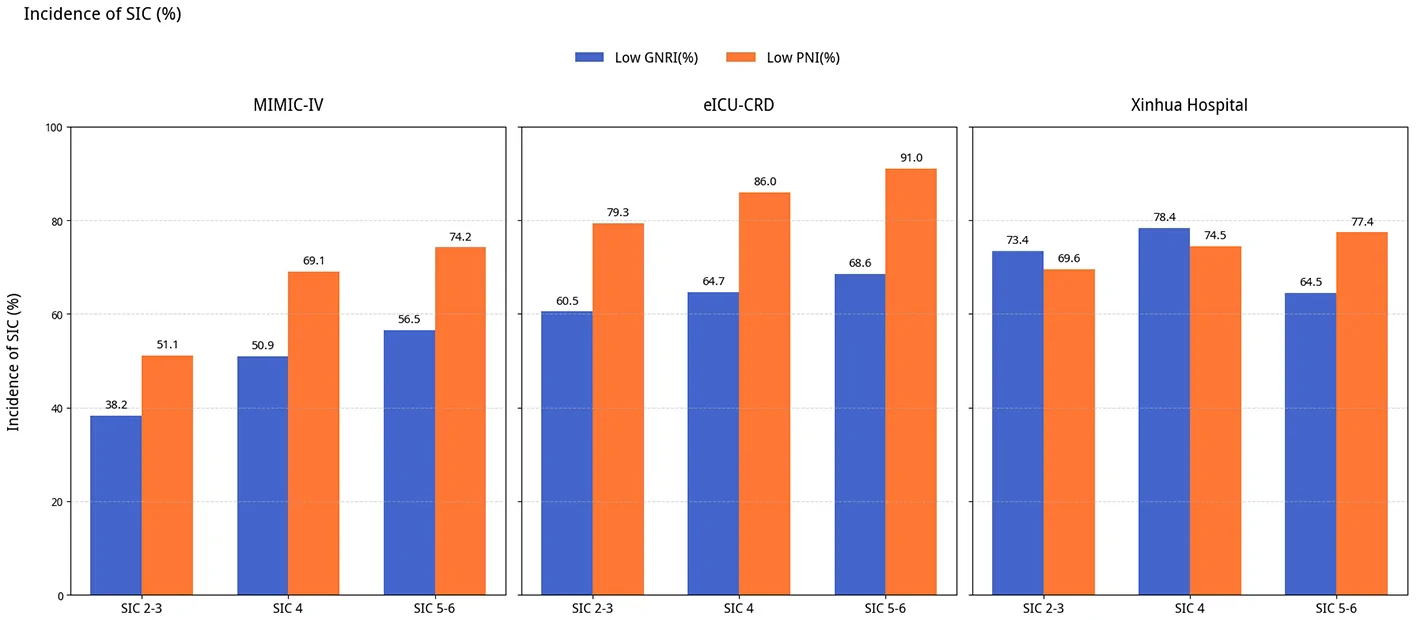
Association between the SIC score and malnutrition in sepsis.

**Figure 3:**
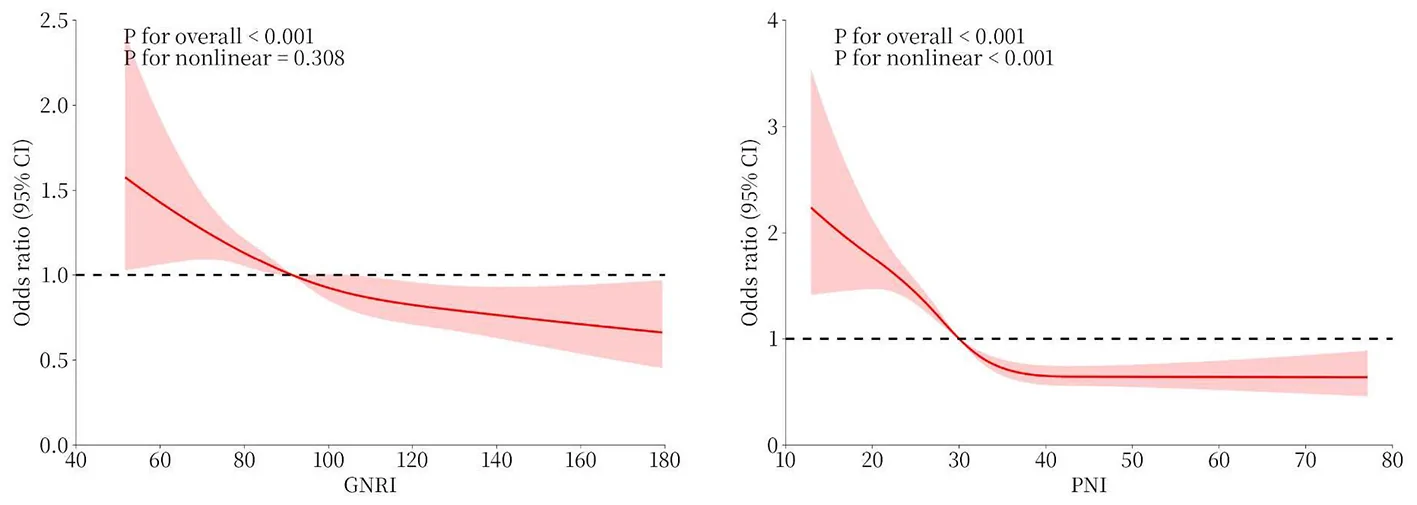
Restricted cubic spline analysis based on logistic regression for the eICU database.

SIC scores from all three cohorts were categorized into three grades (2–3, 4, and 5–6 points) to compare variations in nutritional status across different SIC severity levels. Figure 2 illustrated low nutritional status across different SIC score groups. As SIC scores increased, the prevalence of malnutrition also elevated. Excluding the GNRI from the Xinhua Hospital dataset, the aforementioned findings were not observed in the high SIC score group. Table 3 summarized the results of univariate and multivariate logistic regression analyses used to identify risk factors for SIC based on eICU database. In the univariate logistic regression model, variables with *p* < 0.05 and no multicollinearity (variance inflation factor less than 5) were included in the multivariate logistic regression model. In the multivariate regression model, compared with the group with no nutritional risk, the malnutrition group with both low GNRI and PNI scores (OR 2.18; CI 1.67–2.85; *p* < 0.001) was independently associated with the risk of SIC. This independent association was also validated in the MIMIC-IV cohort (Supplementary Table 5). Although the OR value was greater than 1 in the Xinhua Hospital data, there was no statistical significance (Supplementary Table 6). Figure 3 and Figures 2 and 3 in the Supplementary materials presented the restricted cubic splines generated based on logistic regression analysis from the EICU, MIMIC, and Xinhua Hospital cohorts, respectively. The results demonstrated that patients with higher GNRI and PNI scores were associated with fewer SIC events; in the eICU cohort, the optimal cutoff values were 91.36 for GNRI and 29.99 for PNI.

**Table 3:** Univariate and multivariate logistic regression analysis in the eICU database.

Univariate analysis			Multivariate analysis		
Variables	OR (95%CI)	*P*	Variables	OR (95%CI)	*P*
Nutritional status			Nutritional status		
Both high-group	1.00 (Reference)		Both high-group	1.00 (Reference)	
High GNRI	2.03 (1.56 ~ 2.65)	< 0.001	High GNRI	1.91 (1.42 ~ 2.55)	< 0.001
High PNI	1.34 (0.92 ~ 1.96)	0.129	High PNI	1.35 (0.89 ~ 2.05)	0.155
Both low-group	2.24 (1.76 ~ 2.86)	< 0.001	Both low-group	2.18 (1.67 ~ 2.85)	< 0.001
Hb	0.89 (0.87 ~ 0.92)	< 0.001	Plt	0.99 (0.99 ~ 0.99)	< 0.001
Plt	0.99 (0.99 ~ 0.99)	< 0.001	AST	1.01 (1.01 ~ 1.01)	< 0.001
RDW	1.17 (1.13 ~ 1.20)	< 0.001	TBIL	1.16 (1.10 ~ 1.23)	< 0.001
AST	1.01 (1.01 ~ 1.01)	< 0.001	Lac	1.13 (1.08 ~ 1.18)	< 0.001
TBIL	1.38 (1.29 ~ 1.46)	< 0.001	SOFA	1.05 (1.03 ~ 1.08)	< 0.001
Lac	1.19 (1.15 ~ 1.24)	< 0.001	WBC	1.01 (1.01 ~ 1.02)	0.033
SOFA	1.13 (1.11 ~ 1.15)	< 0.001			
WBC	0.99 (0.98 ~ 0.99)	< 0.001			
Gender					
Female	1.00 (Reference)				
Male	1.01 (0.88 ~ 1.17)	0.858			
Age	1.00 (1.00 ~ 1.01)	0.621			
Height	1.00 (1.00 ~ 1.01)	0.551			
Weight	1.00 (1.00 ~ 1.00)	0.574			
MCV	1.01 (1.00 ~ 1.01)	0.241			
Lymphocytes abs	0.96 (0.93 ~ 1.00)	0.083			
Scr	1.04 (1.00 ~ 1.07)	0.064			
Heart rate	1.00 (1.00 ~ 1.01)	0.141			

Hb, hemoglobin; Plt, platelets; WBC, white blood cell; RDW, red blood cell distribution width; MCV, mean corpuscular volume; AST, aspartate aminotransferase; TBIL, total bilirubin; Scr, iserum creatinine; Lac, lactate; SOFA, sequential organ failure assessment; GNRI, geriatric nutritional risk index; PNI, prognostic nutritional index.

### 3.3 The importance of nutritional indicators in SIC

**Figure 4:**
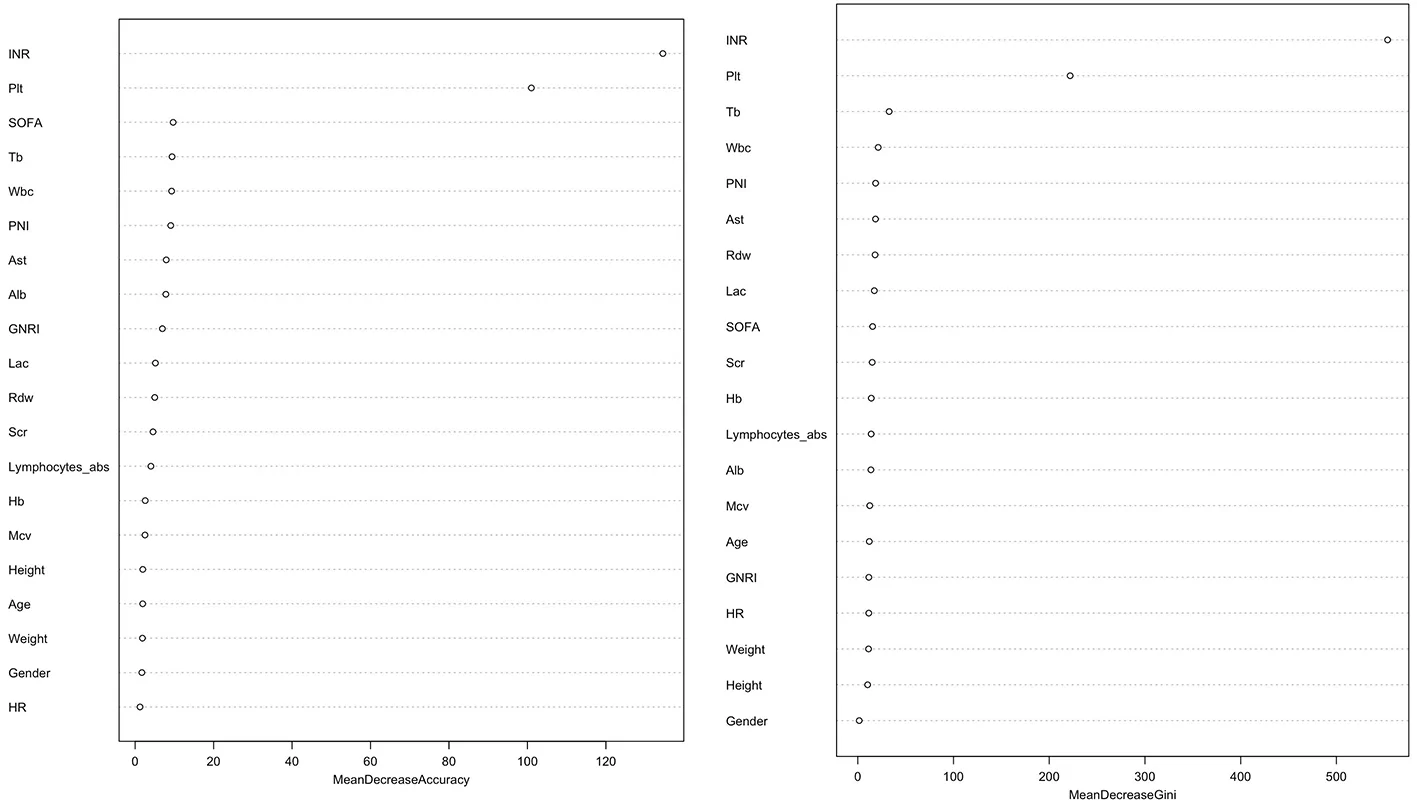
Random forest ranks the importance of factors contributing to SIC.

We used the Random Forest algorithm to build a model for predicting SIC occurrence based on three databases, and calculated the importance rankings of GNRI and PNI among the model's features. Figure 4 and Figures 4 and 5 in the Supplementary materials displayed the importance rankings for the eICU, MIMIC, and Xinhua Hospital datasets, respectively, illustrating the priority of GNRI and PNI. Feature importance was quantified *via* mean decrease in accuracy and mean decrease in Gini coefficient. The results confirmed that GNRI and PNI consistently ranked as top predictive indicators across all three cohorts.

### 3.4 Interaction analysis on additive and multiplicative scales

To further clarify whether the combined effect of GNRI and PNI exceeds the sum of their individual effects, we evaluated their interaction at both the additive and multiplicative levels. In the additive model, we calculated the RERI and the attributable proportion (AP) across three datasets. The 95% confidence intervals for both RERI and AP cross the zero line, indicating that the additive interaction was not statistically significant (Table 4). These findings indicated that the combined risk of reduced GNRI and PNI did not significantly exceed the sum of their individual independent risks. At the multiplicative level, we included an interaction term (GNRI × PNI) in our regression model. In the sensitivity analysis, this interaction term was not statistically significant, indicating that there was no significant multiplicative interaction (Table 4). Collectively, these outcomes demonstrated no significant additive or multiplicative interaction between GNRI and PNI.

**Table 4:** Interaction analysis on additive and multiplicative scales.

Scale type	Interaction analysis metrics	eICU	MIMIC	Xinhua hospital
Additive scale	RERI (95%CI)	−0.188 (−0.488 to 0.173)	−0.02 (−0.38 to 0.448)	0.417 (−0.02 to 1.508)
	AP (95%CI)	−0.311 (−0.844 to 0.29)	−0.029 (−0.567 to 0.678)	1.891 (−0.196 to 20.635)
Multiplicative scale	OR (95%CI)	1 (1−−1.001)	1 (1−−1.001)	1.003 (0.944−−1.009)

RERI, relative excess risk due to interaction; AP, attributable proportion; CI, confidence interval; OR, odds ratio.

### 3.5 Correlations between nutritional indicators and components of the SIC score

**Figure 5:**
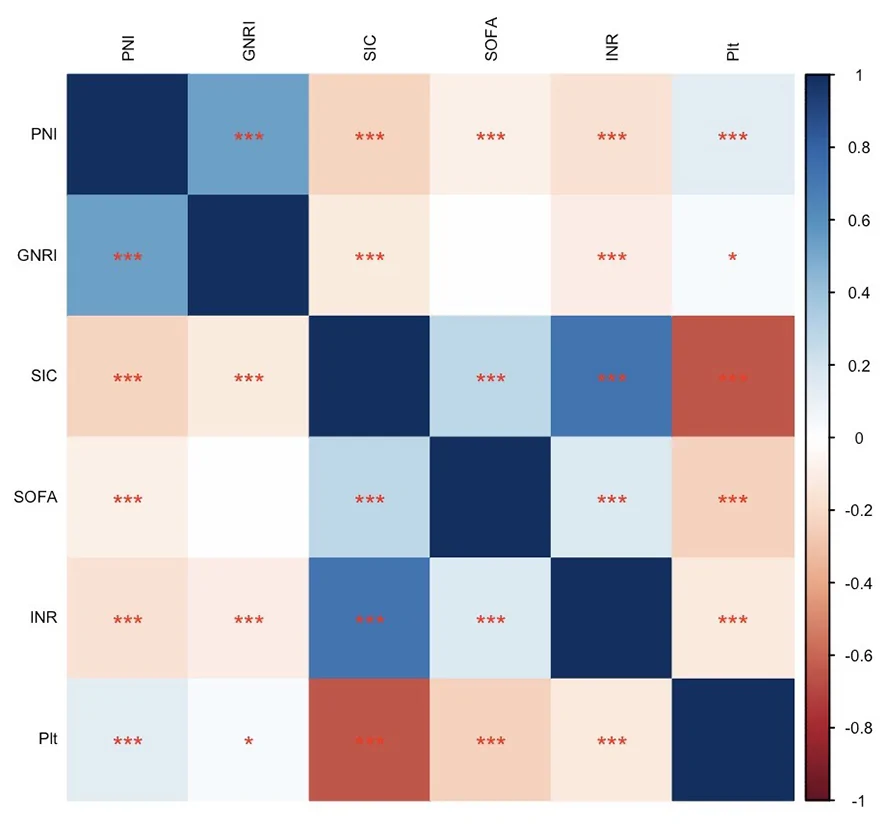
Correlation matrix between nutritional indicators and SIC components in eICU database. The negatively relation is represented in red, and positive relation is represented in blue. The darker the color, the higher the correlation is. *p < 0.05; **p < 0.01; ***p < 0.001.

Spearman correlation analysis was performed to explore the correlations between nutritional markers and individual SIC score components in the eICU, MIMIC-IV, and Xinhua Hospital cohorts, as illustrated in Figure 5 and Supplementary Figures 6 and 7. The same conclusions were drawn from all three cohorts: GNRI is positively correlated with PNI; GNRI and PNI are negatively correlated with SIC scores, SOFA scores, and INR, and positively correlated with platelet count. These findings indicated that superior nutritional status is correlated with a lower risk of sepsis-induced coagulopathy in septic patients.

## 4 Discussion

To our knowledge, this is the first study to investigate the association between nutritional status and SIC in patients with sepsis. The findings demonstrated that, after adjusting for potential confounding factors, nutritional status was independently associated with the risk of SIC in sepsis. Moreover, septic patients with malnutrition complicated by SIC presented a higher 28-day mortality rate.

The pathogenesis of sepsis-induced coagulopathy (SIC) is a complex and multifactorial process mediated by the interplay between systemic inflammation, endothelial dysfunction, and hemostatic imbalance (26). During microbial infection, pathogen-associated molecular patterns activate immune and endothelial cells *via* Toll-like receptors, triggering a massive release of pro-inflammatory cytokines such as tumor necrosis factor-alpha and interleukin-6 (27). These cytokines upregulate tissue factor expression on monocytes and endothelial cells, initiating the extrinsic coagulation cascade and generating thrombin (28). Concurrently, natural anticoagulant pathways are severely compromised: antithrombin levels are depleted, while the protein C system is inhibited by downregulation of endothelial thrombomodulin and endothelial protein C receptor caused by inflammatory mediators (29). Furthermore, fibrinolysis is suppressed primarily through a marked increase in plasminogen activator inhibitor-1 released from activated platelets and endothelial cells, shifting the balance toward fibrin deposition (5, 27). Platelets are also directly activated by thrombin, cytokines, and neutrophil extracellular traps, promoting aggregation and microthrombi formation. The resulting widespread microvascular thrombosis leads to tissue hypoperfusion, multi-organ dysfunction, and ultimately disseminated intravascular coagulation.

To date, these nutritional indicators have been extensively studied in other fields. The GNRI is a simple and effective tool for assessing the risk of malnutrition in older adults and predicting clinical outcomes. Previous studies have shown that, in the management of sepsis patients across all age groups, GNRI and PNI scores may help identify patients at high risk of death (18). In various clinical settings, including trauma and acute kidney injury, higher GNRI scores have been associated with improved outcomes (30, 31). Conversely, in conditions such as cancer and infective endocarditis, lower PNI scores have been linked to an increased risk of poor outcomes (32, 33). The relationship between malnutrition and SIC has gained increasing attention, as malnutrition can exacerbate coagulation disorders in septic patients through multiple pathways. First, protein-energy malnutrition impairs hepatic synthetic function, leading to reduced production of natural anticoagulants such as antithrombin (AT), protein C (PC), and protein S, thereby weakening the body's innate anticoagulant capacity (26). Second, vitamin K deficiency is common in malnourished patients, and vitamin K is an essential cofactor for the synthesis of coagulation factors II, VII, IX, X as well as PC and PS; deficiency can lead to reduced activity of both procoagulant and anticoagulant factors, resulting in complex hemostatic abnormalities (34). In addition, deficiencies in vitamins and trace elements such as zinc and selenium can impair antioxidant defenses and immune function, leading to uncontrolled inflammation, which in turn upregulates tissue factor expression, inhibits fibrinolysis, and promotes microthrombosis (35). Clinical studies have also shown that malnourished patients have a significantly higher risk of organ dysfunction and higher in-hospital mortality (36). Therefore, malnutrition is not only a risk factor for poor prognosis in patients with sepsis but also directly contributes to the pathogenesis of SIC by impairing the anticoagulant system, exacerbating inflammation, and promoting coagulation activation. This underscores the importance of early nutritional assessment and intervention in improving coagulation outcomes.

Data from the MIMIC database were collected from 2008 to 2022, while data from the eICU database were collected from 2014 to 2015; the study populations in both databases consisted primarily of White and Black individuals. The Xinhua Hospital dataset comprised Chinese individuals from 2021 to 2022. Despite the heterogeneity in study periods, ethnic backgrounds, and regional populations across the three datasets, the core study conclusion remained robust: malnutrition is closely correlated with an increased incidence of SIC in septic patients. However, due to the small sample size in the Xinhua Hospital study, no statistically significant differences were observed. Notably, significant baseline differences in GNRI and PNI values existed among the three cohorts, indicating that localized threshold calibration is required before the widespread application of these nutritional markers in different regional ICU populations.

This study had several limitations. Although multicenter database resources were adopted for analysis, the retrospective study design inevitably introduced selection bias, and the final model failed to incorporate all potential confounding variables. We failed to exclude patients with underlying conditions that might interfere with the calculation of nutritional indicators, such as hematological malignancies. Additionally, preoperative albumin and nutritional supplementation records were not collected in this study. Although our Xinhua Hospital cohort exhibited trends similar to those observed in the other two cohorts, no significant statistical differences were found, likely due to limitations in sample size and statistical power. We need more local data to support this study. There are numerous diagnostic criteria for assessing nutritional status and SIC. Although we used the most common diagnostic criteria for ISTH-SIC and two nutritional indicators to jointly assess nutritional status, misdiagnosis of SIC and nutritional status remains inevitable. This study did not include long-term survival data and was therefore unable to examine the relationship between long-term prognosis and nutritional status and SIC. Finally, as this study employed a retrospective design, further prospective studies are needed to focus on the association between nutritional status and SIC.

## 5 Conclusion

In summary, malnutrition is associated with a higher incidence of SIC. Sepsis patients with SIC or malnutrition have a higher 28-day mortality rate.

## Data Availability

The datasets used and analyzed in this study are available upon request from the corresponding author.
