# Supplementary material for: Association between nutritional status and sepsis-induced coagulopathy: a multicenter retrospective cohort study
Source: Front Nutr. 2026 Aug 10;13:1894405. doi: 10.3389/fnut.2026.1894405 (PMC13500337; doi:10.3389/fnut.2026.1894405)
Supplement: Supplementary file 1 [file Table_1.docx]

Figure S1: MIMIC-IV database screening flowchart

Table S1: Baseline information in the MIMIC-IVdatabase, grouped by the occurrence of SIC

| Variables | Total (n = 1466) | No-SIC (n = 606) | SIC (n = 860) | P |
| --- | --- | --- | --- | --- |
| Age (years) | 64.65 (53.00, 75.44) | 66.88 (54.76, 76.81) | 63.39 (52.02, 74.12) | <.001 |
| Gender, n(%) |  |  |  | 0.519 |
| Female | 600 (40.93) | 254 (41.91) | 346 (40.23) |  |
| Male | 866 (59.07) | 352 (58.09) | 514 (59.77) |  |
| Height (cm) | 170.00 (163.00, 178.00) | 170.00 (163.00, 178.00) | 170.00 (163.00, 178.00) | 0.273 |
| Weight (kg) | 81.25 (68.10, 97.70) | 82.30 (68.03, 98.90) | 80.70 (68.10, 96.90) | 0.441 |
| Body Mass Index (kg/m2) | 28.07 (24.19, 33.33) | 28.41 (24.31, 33.93) | 27.57 (24.13, 32.89) | 0.157 |
| Heart rate (bpm) | 94.00 (81.00, 110.00) | 92.00 (80.00, 107.00) | 97.00 (81.75, 111.00) | <.001 |
| Lymphocyte count (×10^9/L) | 0.93 (0.55, 1.49) | 1.03 (0.63, 1.63) | 0.84 (0.50, 1.38) | <.001 |
| Hb (g/dL) | 10.40 (8.80, 12.30) | 11.35 (9.60, 12.90) | 9.90 (8.30, 11.60) | <.001 |
| Plt (×10^9/L) | 165.50 (101.00, 249.00) | 219.00 (166.50, 285.00) | 117.50 (70.75, 191.00) | <.001 |
| WBC (×10^9/L) | 12.80 (8.00, 19.00) | 13.90 (9.70, 19.67) | 11.70 (7.00, 18.60) | <.001 |
| RDW (%) | 15.40 (14.10, 17.40) | 14.55 (13.50, 15.90) | 16.10 (14.60, 18.20) | <.001 |
| MCV (fL) | 93.00 (88.00, 98.00) | 92.00 (88.00, 96.00) | 94.00 (89.00, 100.00) | <.001 |
| Alb (g/dL) | 3.00 (2.50, 3.40) | 3.20 (2.70, 3.60) | 2.80 (2.40, 3.30) | <.001 |
| ALT (U/L) | 40.00 (20.00, 138.00) | 32.50 (18.00, 81.75) | 49.00 (23.00, 214.75) | <.001 |
| TBIL (mg/dL) | 1.00 (0.50, 2.59) | 0.60 (0.40, 1.10) | 1.90 (0.80, 4.80) | <.001 |
| INR | 1.40 (1.20, 1.90) | 1.20 (1.10, 1.30) | 1.80 (1.50, 2.40) | <.001 |
| Scr (mg/dL) | 1.50 (0.90, 2.50) | 1.40 (0.90, 2.20) | 1.60 (1.00, 2.70) | <.001 |
| SOFA Score | 4.00 (3.00, 6.00) | 4.00 (2.00, 5.00) | 5.00 (3.00, 7.00) | <.001 |
| GNRI | 99.03 (87.58, 111.09) | 102.42 (91.08, 114.37) | 96.42 (85.58, 108.12) | <.001 |
| PNI | 35.40 (29.49, 40.72) | 37.80 (32.43, 43.16) | 33.70 (28.25, 38.85) | <.001 |
| History of liver disease, n(%) |  |  |  | <.001 |
| No | 1177 (80.29) | 584 (96.37) | 593 (68.95) |  |
| Yes | 289 (19.71) | 22 (3.63) | 267 (31.05) |  |
| History of kidney disease, n(%) |  |  |  | 0.511 |
| No | 1092 (74.49) | 446 (73.60) | 646 (75.12) |  |
| Yes | 374 (25.51) | 160 (26.40) | 214 (24.88) |  |
| Malignant Cancer, n(%) |  |  |  | 0.020 |
| No | 1252 (85.40) | 533 (87.95) | 719 (83.60) |  |
| Yes | 214 (14.60) | 73 (12.05) | 141 (16.40) |  |
| Diabetes, n(%) |  |  |  | 0.345 |
| No | 1007 (68.69) | 408 (67.33) | 599 (69.65) |  |
| Yes | 459 (31.31) | 198 (32.67) | 261 (30.35) |  |
| Length of hospital stay (days) | 13.00 (7.00, 22.00) | 12.00 (7.00, 21.00) | 13.00 (7.00, 23.00) | 0.461 |
| Length of ICU stay (days) | 5.00 (3.00, 11.00) | 6.00 (3.00, 12.00) | 5.00 (3.00, 10.00) | 0.013 |
| Outcome28d, n(%) |  |  |  | <.001 |
| Alive | 1007 (68.69) | 459 (75.74) | 548 (63.72) |  |
| Death | 459 (31.31) | 147 (24.26) | 312 (36.28) |  |

Abbreviations: hemoglobin(Hb), platelets(Plt), white blood cell (WBC), red blood cell distribution Width(RDW), mean corpuscular volume(MCV), albumin(Alb), alanine aminotransferase(ALT), total bilirubin (TBIL), international normalization ratio(INR), serum creatinine(Scr), Sequential Organ Failure Assessment(SOFA), Geriatric Nutritional Risk Index (GNRI), Prognostic Nutritional Index (PNI).

Table S2: Baseline information in the Xinhua Hospital database, grouped by the occurrence of SIC

| **Variables** | **Total (n = 214)** | **No-SIC (n = 132)** | **SIC (n = 82)** | **P** |
| --- | --- | --- | --- | --- |
| Age (years) | 72.00 (66.25, 80.00) | 72.00 (67.00, 81.00) | 72.00 (65.00, 80.00) | 0.475 |
| Gender, n(%) |  |  |  | 0.890 |
| Female | 90 (42.06) | 56 (42.42) | 34 (41.46) |  |
| Male | 124 (57.94) | 76 (57.58) | 48 (58.54) |  |
| Height (cm) | 168.00 (160.00, 172.00) | 166.50 (160.00, 172.00) | 168.00 (160.00, 173.00) | 0.406 |
| Weight (kg) | 70.00 (60.00, 75.00) | 68.50 (59.25, 75.00) | 70.00 (60.00, 75.00) | 0.406 |
| Body Mass Index (kg/m^2^) | 24.22 (21.78, 26.66) | 24.03 (21.74, 26.63) | 24.47 (21.81, 26.84) | 0.617 |
| Heart rate (bpm) | 93.00 (82.00, 103.00) | 91.00 (80.00, 102.00) | 95.50 (83.25, 110.25) | 0.021 |
| Lymphocyte count (×10^9/L) | 0.76 (0.48, 1.08) | 0.79 (0.53, 1.08) | 0.67 (0.40, 1.08) | 0.216 |
| Hb (g/L) | 117.00 (103.00, 129.00) | 118.00 (104.50, 129.00) | 115.50 (100.25, 129.00) | 0.797 |
| Plt (×10^9/L) | 143.00 (102.00, 195.75) | 176.50 (131.50, 214.50) | 97.50 (65.25, 134.75) | <.001 |
| WBC (×10^9/L) | 10.18 (7.29, 14.87) | 9.70 (7.07, 13.44) | 10.87 (7.65, 16.70) | 0.156 |
| RDW (%) | 13.40 (12.90, 14.30) | 13.20 (12.70, 14.15) | 13.50 (13.10, 14.50) | 0.021 |
| MCHC (g/L) | 336.00 (328.25, 343.00) | 335.00 (327.00, 341.00) | 337.50 (331.00, 345.00) | 0.034 |
| Alb (g/L) | 30.60 (28.22, 33.60) | 30.85 (28.48, 34.32) | 29.85 (27.80, 32.70) | 0.112 |
| ALT (U/L) | 26.50 (16.00, 58.00) | 24.00 (14.00, 49.50) | 30.50 (20.00, 82.50) | 0.002 |
| TBIL (μmol/L) | 15.50 (10.50, 22.08) | 13.25 (9.30, 18.50) | 19.00 (14.20, 26.85) | <.001 |
| INR | 1.15 (1.04, 1.30) | 1.10 (1.03, 1.18) | 1.30 (1.17, 1.44) | <.001 |
| Scr (μmol/L) | 91.75 (67.53, 151.02) | 84.00 (61.08, 131.62) | 117.45 (80.33, 198.57) | 0.001 |
| SOFA Score | 4.00 (2.00, 6.00) | 3.00 (2.00, 5.00) | 5.00 (3.00, 8.00) | <.001 |
| GNRI | 92.23 (85.27, 98.67) | 93.30 (86.02, 98.69) | 88.96 (84.79, 98.56) | 0.401 |
| PNI | 34.38 (31.38, 38.39) | 35.67 (31.55, 39.27) | 33.88 (31.22, 37.50) | 0.100 |
| Hypertension, n(%) |  |  |  | 0.150 |
| No | 81 (37.85) | 45 (34.09) | 36 (43.90) |  |
| Yes | 133 (62.15) | 87 (65.91) | 46 (56.10) |  |
| Diabetes, n(%) |  |  |  | 0.692 |
| No | 119 (55.61) | 72 (54.55) | 47 (57.32) |  |
| Yes | 95 (44.39) | 60 (45.45) | 35 (42.68) |  |
| Length of hospital stay (days) | 9.00 (6.00, 13.00) | 9.00 (6.00, 12.00) | 9.00 (7.00, 14.00) | 0.227 |
| Outcome28d, n(%) |  |  |  | 0.259 |
| Alive | 194 (90.65) | 122 (92.42) | 72 (87.80) |  |
| Death | 20 (9.35) | 10 (7.58) | 10 (12.20) |  |

Abbreviations: hemoglobin(Hb), platelets(Plt), white blood cell (WBC), red blood cell distribution Width(RDW), mean corpuscular hemoglobin concentration(MCHC), albumin(Alb), alanine aminotransferase(ALT), total bilirubin (TBIL), international normalization ratio(INR), serum creatinine(Scr), Sequential Organ Failure Assessment(SOFA), Geriatric Nutritional Risk Index (GNRI), Prognostic Nutritional Index (PNI).

Table S3: Baseline information in the MIMIC-IV database, grouped by the occurrence of nutritional risk

| **Variables** | **Both high-group (n = 419)** | **High GNRI (n = 352)** | **High PNI (n = 120)** | **Both low-group (n = 575)** | **P** |
| --- | --- | --- | --- | --- | --- |
| Age (years) | 64.60 (53.35,75.34) | 65.08 (52.49,74.13) | 63.55 (51.90,78.68) | 64.52 (53.24,76.41) | 0.694 |
| Gender, n(%) |  |  |  |  | 0.046 |
| Female | 149 (35.56) | 154 (43.75) | 56 (46.67) | 241 (41.91) |  |
| Male | 270 (64.44) | 198 (56.25) | 64 (53.33) | 334 (58.09) |  |
| Weight (kg) | 90.00 (78.35,106.35) | 99.00 (87.88,115.32) | 62.35 (55.38,70.00) | 70.30 (61.00,81.00) | <.001 |
| Height (cm) | 170.00 (165.00,178.00) | 168.00 (160.00,178.00) | 168.00 (163.00,174.25) | 170.00 (163.00,175.00) | 0.002 |
| Body Mass Index (kg/m2) | 30.43 (27.05,35.95) | 34.89 (31.63,40.31) | 22.03 (19.88,24.23) | 24.89 (22.05,27.43) | <.001 |
| Heart rate (bpm) | 89.00 (78.00,108.00) | 96.00 (83.00,110.00) | 90.00 (78.00,105.00) | 97.00 (83.00,110.50) | <.001 |
| Lymphocyte count (×10^9/L) | 1.38 (0.84,1.96) | 0.74 (0.44,1.07) | 1.71 (1.03,2.49) | 0.73 (0.41,1.16) | <.001 |
| Hb (g/dL) | 11.80 (9.80,13.40) | 10.50 (8.90,12.03) | 10.60 (9.07,12.20) | 9.70 (8.20,11.20) | <.001 |
| Plt (×10^9/L) | 186.00 (135.00,257.50) | 159.50 (95.75,234.75) | 185.50 (114.50,261.50) | 146.00 (87.00,239.00) | <.001 |
| WBC (×10^9/L) | 13.50 (9.25,19.35) | 12.80 (7.60,18.85) | 15.65 (10.30,21.88) | 11.80 (7.20,18.45) | <.001 |
| RDW (%) | 14.80 (13.55,17.00) | 15.60 (14.40,17.60) | 15.15 (14.00,17.02) | 15.80 (14.40,17.70) | <.001 |
| MCV (fL) | 92.60 (88.00,97.00) | 93.00 (88.00,98.00) | 94.00 (88.75,100.00) | 93.00 (89.00,98.00) | 0.116 |
| Alb (g/dL) | 3.70 (3.40,4.00) | 2.90 (2.60,3.10) | 3.30 (2.90,3.60) | 2.50 (2.20,2.80) | <.001 |
| Scr (mg/dL) | 1.50 (1.00,2.55) | 1.70 (1.10,2.62) | 1.40 (0.90,2.10) | 1.30 (0.80,2.30) | <.001 |
| INR | 1.30 (1.10,1.70) | 1.40 (1.20,1.90) | 1.40 (1.20,1.90) | 1.50 (1.30,2.00) | <.001 |
| TBIL (mg/dL) | 0.80 (0.50,2.10) | 1.40 (0.50,3.32) | 0.90 (0.50,2.57) | 1.10 (0.50,2.95) | <.001 |
| ALT (U/L) | 35.00 (19.50,121.00) | 51.00 (24.75,164.00) | 36.50 (17.00,131.50) | 39.00 (20.00,135.50) | 0.021 |
| SOFA Score | 4.00 (3.00,6.00) | 4.00 (3.00,7.00) | 4.00 (2.00,6.00) | 4.00 (3.00,7.00) | <.001 |
| PNI | 43.75 (40.50,47.20) | 33.70 (29.76,35.99) | 40.70 (39.35,43.95) | 29.58 (25.85,33.50) | <.001 |
| GNRI | 112.79 (105.37,123.04) | 108.28 (101.91,117.60) | 91.79 (87.14,95.05) | 85.83 (78.90,91.30) | <.001 |
| SIC Score | 3.00 (2.00,4.00) | 4.00 (3.00,5.00) | 4.00 (2.00,5.00) | 4.00 (3.00,5.00) | <.001 |
| Diabetes, n(%) |  |  |  |  | <.001 |
| No | 268 (63.96) | 210 (59.66) | 93 (77.50) | 436 (75.83) |  |
| Yes | 151 (36.04) | 142 (40.34) | 27 (22.50) | 139 (24.17) |  |
| History of kidney disease, n(%) |  |  |  |  | 0.454 |
| No | 302 (72.08) | 262 (74.43) | 88 (73.33) | 440 (76.52) |  |
| Yes | 117 (27.92) | 90 (25.57) | 32 (26.67) | 135 (23.48) |  |
| Malignant Cancer, n(%) |  |  |  |  | 0.010 |
| No | 374 (89.26) | 301 (85.51) | 106 (88.33) | 471 (81.91) |  |
| Yes | 45 (10.74) | 51 (14.49) | 14 (11.67) | 104 (18.09) |  |
| History of liver disease, n(%) |  |  |  |  | 0.218 |
| No | 347 (82.82) | 276 (78.41) | 101 (84.17) | 453 (78.78) |  |
| Yes | 72 (17.18) | 76 (21.59) | 19 (15.83) | 122 (21.22) |  |
| Length of hospital stay (days) | 11.00 (6.00,20.00) | 13.00 (7.00,22.00) | 12.00 (6.00,21.00) | 14.00 (7.00,23.00) | 0.060 |
| Length of ICU stay (days) | 5.63 (3.00,10.00) | 6.00 (3.00,12.00) | 5.00 (3.00,9.00) | 5.00 (3.00,10.00) | 0.154 |
| Outcome28d, n(%) |  |  |  |  | 0.098 |
| Alive | 306 (73.03) | 237 (67.33) | 85 (70.83) | 379 (65.91) |  |
| Death | 113 (26.97) | 115 (32.67) | 35 (29.17) | 196 (34.09) |  |
| SIC, n(%) |  |  |  |  | <.001 |
| No | 243 (58.00) | 131 (37.22) | 53 (44.17) | 179 (31.13) |  |
| Yes | 176 (42.00) | 221 (62.78) | 67 (55.83) | 396 (68.87) |  |

Abbreviations: hemoglobin(Hb), platelets(Plt), white blood cell (WBC), red blood cell distribution Width(RDW), mean corpuscular volume(MCV), albumin(Alb), alanine aminotransferase(ALT), total bilirubin (TBIL), international normalization ratio(INR), serum creatinine(Scr), Sequential Organ Failure Assessment(SOFA), Geriatric Nutritional Risk Index (GNRI), Prognostic Nutritional Index (PNI), sepsis-induced coagulopathy (SIC).

Table S4: Baseline information in the Xinhua Hospital database, grouped by the occurrence of nutritional risk

| **Variables** | **Both high-group (n = 37)** | **High GNRI (n = 20)** | **High PNI (n = 23)** | **Both low-group (n = 134)** | **P** |
| --- | --- | --- | --- | --- | --- |
| Age (years) | 70.00 (66.00,74.00) | 75.50 (68.25,79.25) | 78.00 (70.00,85.00) | 71.50 (66.25,80.75) | 0.070 |
| Gender, n(%) |  |  |  |  | 0.032 |
| Female | 18 (48.65) | 14 (70.00) | 8 (34.78) | 50 (37.31) |  |
| Male | 19 (51.35) | 6 (30.00) | 15 (65.22) | 84 (62.69) |  |
| Weight (kg) | 72.50 (65.00,80.00) | 75.00 (70.75,82.12) | 62.00 (55.00,65.00) | 65.00 (55.25,75.00) | <.001 |
| Height (cm) | 165.00 (160.00,168.00) | 160.00 (157.75,165.25) | 170.00 (161.00,172.00) | 170.00 (160.00,174.00) | 0.006 |
| Body Mass Index (kg/m^2^) | 26.56 (23.73,28.91) | 29.26 (27.54,31.45) | 21.80 (20.80,23.83) | 23.37 (20.98,25.70) | <.001 |
| Heart rate (bpm) | 92.00 (81.00,102.00) | 88.00 (75.75,102.25) | 91.00 (79.50,95.00) | 95.00 (84.00,107.00) | 0.095 |
| Lymphocyte count (×10^9/L) | 1.08 (0.85,1.29) | 0.66 (0.38,1.00) | 1.09 (0.78,1.44) | 0.63 (0.40,0.94) | <.001 |
| WBC (×10^9/L) | 10.19 (8.53,13.32) | 11.80 (7.32,15.20) | 8.49 (5.54,10.90) | 10.24 (7.39,15.47) | 0.469 |
| MCHC (g/L) | 336.00 (329.00,344.00) | 338.50 (330.75,340.25) | 333.00 (326.50,339.50) | 335.50 (329.00,343.00) | 0.542 |
| RDW (%) | 13.00 (12.40,13.90) | 13.10 (12.73,13.93) | 13.50 (12.90,14.05) | 13.50 (13.00,14.50) | 0.062 |
| Alb (g/L) | 36.80 (34.30,39.00) | 31.25 (30.37,33.00) | 34.50 (32.80,35.70) | 28.85 (27.13,30.78) | <.001 |
| INR | 1.15 (1.04,1.30) | 1.14 (1.07,1.25) | 1.09 (1.03,1.25) | 1.17 (1.05,1.30) | 0.560 |
| Scr (μmol/L) | 84.00 (62.40,134.00) | 106.25 (76.30,178.92) | 83.80 (66.75,102.10) | 100.00 (68.35,158.57) | 0.258 |
| TBIL (μmol/L) | 16.60 (10.60,29.70) | 15.65 (10.00,27.23) | 15.10 (10.40,18.75) | 15.25 (10.62,20.80) | 0.428 |
| ALT (U/L) | 30.00 (22.00,75.00) | 50.50 (24.50,69.00) | 15.00 (9.50,26.00) | 26.00 (16.00,58.00) | 0.003 |
| Hb (g/L) | 125.00 (115.00,140.00) | 117.00 (100.75,131.50) | 116.00 (102.00,127.50) | 114.50 (100.00,128.00) | 0.017 |
| Plt (×10^9/L) | 150.00 (107.00,198.00) | 112.00 (95.50,154.00) | 179.00 (140.00,200.00) | 139.00 (101.00,184.75) | 0.051 |
| SOFA Score | 3.00 (2.00,5.00) | 4.50 (3.00,7.00) | 3.00 (2.00,4.00) | 4.00 (3.00,7.00) | 0.015 |
| GNRI | 105.00 (102.15,109.48) | 101.91 (99.57,106.86) | 94.71 (89.03,95.55) | 87.56 (81.45,92.43) | <.001 |
| PNI | 41.95 (39.50,44.35) | 34.90 (33.84,36.10) | 39.50 (39.15,40.58) | 32.35 (30.04,34.39) | <.001 |
| SIC | 3.00 (2.00,4.00) | 3.00 (3.00,5.00) | 3.00 (2.00,4.00) | 3.00 (3.00,4.00) | 0.141 |
| Diabetes, n(%) |  |  |  |  | 0.159 |
| No | 21 (56.76) | 8 (40.00) | 17 (73.91) | 73 (54.48) |  |
| Yes | 16 (43.24) | 12 (60.00) | 6 (26.09) | 61 (45.52) |  |
| Hypertension, n(%) |  |  |  |  | 0.298 |
| No | 10 (27.03) | 6 (30.00) | 11 (47.83) | 54 (40.30) |  |
| Yes | 27 (72.97) | 14 (70.00) | 12 (52.17) | 80 (59.70) |  |
| Length of hospital stay (days) | 9.00 (6.00,11.00) | 9.00 (7.00,11.75) | 7.00 (5.50,8.50) | 9.00 (7.00,14.00) | 0.040 |
| Outcome28d, n(%) |  |  |  |  | 0.526 |
| Alive | 36 (97.30) | 18 (90.00) | 21 (91.30) | 119 (88.81) |  |
| Death | 1 (2.70) | 2 (10.00) | 2 (8.70) | 15 (11.19) |  |
| SIC, n(%) |  |  |  |  | 0.746 |
| No | 24 (64.86) | 11 (55.00) | 16 (69.57) | 81 (60.45) |  |
| Yes | 13 (35.14) | 9 (45.00) | 7 (30.43) | 53 (39.55) |  |

Abbreviations: hemoglobin(Hb), platelets(Plt), white blood cell (WBC), red blood cell distribution Width(RDW), mean corpuscular hemoglobin concentration(MCHC), albumin(Alb), alanine aminotransferase(ALT), total bilirubin (TBIL), international normalization ratio(INR), serum creatinine(Scr), Sequential Organ Failure Assessment(SOFA), Geriatric Nutritional Risk Index (GNRI), Prognostic Nutritional Index (PNI), sepsis-induced coagulopathy (SIC).

Table S5: Univariate and multivariate logistic regression analysis in the MIMIC-IV database

| **Univariate analysis** | | | **Multivariate analysis** | | |
| --- | --- | --- | --- | --- | --- |
| **Variables** | **OR (95%CI)** | P | **Variables** | **OR (95%CI)** | P |
| Nutritional Status |  |  | Nutritional Status |  |  |
| Both high-group | 1.00 (Reference) |  | Both high-group | 1.00 (Reference) |  |
| High GNRI | 2.33 (1.74 ~ 3.11) | <.001 | High GNRI | 1.67 (1.16 ~ 2.39) | 0.005 |
| High PNI | 1.75 (1.16 ~ 2.63) | 0.008 | High PNI | 1.70 (1.02 ~ 2.84) | 0.04 |
| Both low-group | 3.05 (2.35 ~ 3.97) | <.001 | Both low-group | 2.21 (1.58 ~ 3.11) | <.001 |
| Age | 0.99 (0.98 ~ 0.99) | 0.001 | Age | 1.00 (0.99 ~ 1.01) | 0.532 |
| Lymphocytes abs | 0.98 (0.92 ~ 1.03) | 0.414 | Hb | 0.87 (0.82 ~ 0.92) | <.001 |
| Hb | 0.79 (0.76 ~ 0.83) | <.001 | Plt | 0.99 (0.99 ~ 0.99) | <.001 |
| Plt | 0.99 (0.99 ~ 0.99) | <.001 | WBC | 1.01 (1.00 ~ 1.02) | 0.166 |
| WBC | 0.99 (0.98 ~ 0.99) | 0.015 | RDW | 1.16 (1.09 ~ 1.23) | <.001 |
| RDW | 1.31 (1.25 ~ 1.38) | <.001 | MCV | 1.01 (0.99 ~ 1.02) | 0.54 |
| MCV | 1.03 (1.02 ~ 1.05) | <.001 | Scr | 0.95 (0.87 ~ 1.04) | 0.278 |
| Scr | 1.12 (1.05 ~ 1.20) | 0.001 | TBIL | 1.19 (1.10 ~ 1.28) | <.001 |
| TBIL | 1.49 (1.38 ~ 1.62) | <.001 | ALT | 1.01 (1.01 ~ 1.01) | <.001 |
| ALT | 1.01 (1.01 ~ 1.01) | <.001 | SOFA | 1.19 (1.11 ~ 1.27) | <.001 |
| SOFA | 1.38 (1.30 ~ 1.45) | <.001 | Heart rate | 1.01 (1.01 ~ 1.01) | 0.041 |
| Weight | 1.00 (1.00 ~ 1.00) | 0.712 |  |  |  |
| Height | 1.01 (1.00 ~ 1.02) | 0.315 |  |  |  |
| Heart rate | 1.01 (1.01 ~ 1.01) | <.001 |  |  |  |
| Gender |  |  |  |  |  |
| Famale | 1.00 (Reference) |  |  |  |  |
| Male | 1.07 (0.87 ~ 1.32) | 0.519 |  |  |  |

Abbreviations: hemoglobin(Hb), platelets(Plt), white blood cell (WBC), red blood cell distribution Width(RDW), mean corpuscular volume(MCV), alanine aminotransferase(ALT), total bilirubin (TBIL), serum creatinine(Scr), Sequential Organ Failure Assessment(SOFA), Geriatric Nutritional Risk Index (GNRI), Prognostic Nutritional Index (PNI), acute kidney injury (AKI).

Table S6: Univariate and multivariate logistic regression analysis in the Xinhua Hospital database

| **Univariate analysis** | | | **Multivariate analysis** | | |
| --- | --- | --- | --- | --- | --- |
| **Variables** | **OR (95%CI)** | **P** | **Variables** | **OR (95%CI)** | **P** |
| Nutritional Status |  |  | Nutritional Status |  |  |
| Both high-group | 1.00 (Reference) |  | Both high-group | 1.00 (Reference) |  |
| High GNRI | 1.51 (0.50 ~ 4.58) | 0.466 | High GNRI | 0.83 (0.19 ~ 3.62) | 0.800 |
| High PNI | 0.81 (0.26 ~ 2.46) | 0.707 | High PNI | 1.92 (0.47 ~ 7.89) | 0.367 |
| Both low-group | 1.21 (0.57 ~ 2.58) | 0.625 | Both low-group | 1.36 (0.48 ~ 3.81) | 0.562 |
| Heart rate | 1.02 (1.01 ~ 1.04) | 0.004 | TBIL | 1.04 (1.01 ~ 1.07) | 0.043 |
| PO2 | 1.00 (1.00 ~ 1.01) | 0.344 | Alt | 1.00 (1.00 ~ 1.01) | 0.080 |
| Lymphocytes abs | 0.82 (0.46 ~ 1.47) | 0.503 | SOFA | 1.04 (0.93 ~ 1.16) | 0.502 |
| WBC | 1.03 (0.99 ~ 1.06) | 0.135 | Plt | 0.98 (0.97 ~ 0.98) | <.001 |
| MCHC | 1.03 (1.01 ~ 1.05) | 0.018 |  |  |  |
| RDW | 1.15 (0.95 ~ 1.38) | 0.141 |  |  |  |
| RBC | 0.83 (0.55 ~ 1.25) | 0.374 |  |  |  |
| Lac | 1.17 (0.99 ~ 1.39) | 0.062 |  |  |  |
| BUN | 1.01 (0.99 ~ 1.03) | 0.269 |  |  |  |
| Scr | 1.00 (1.00 ~ 1.00) | 0.739 |  |  |  |
| TBIL | 1.06 (1.03 ~ 1.09) | <.001 |  |  |  |
| AST | 1.00 (1.00 ~ 1.00) | 0.099 |  |  |  |
| ALT | 1.01 (1.01 ~ 1.01) | 0.009 |  |  |  |
| Weight | 1.01 (0.99 ~ 1.03) | 0.578 |  |  |  |
| Height | 1.01 (0.98 ~ 1.05) | 0.503 |  |  |  |
| SOFA | 1.18 (1.08 ~ 1.28) | <.001 |  |  |  |
| Plt | 0.98 (0.97 ~ 0.98) | <.001 |  |  |  |
| Hb | 1.00 (0.99 ~ 1.01) | 0.834 |  |  |  |
| Gender |  |  |  |  |  |
| Famale | 1.00 (Reference) |  |  |  |  |
| Male | 1.04 (0.59 ~ 1.82) | 0.89 |  |  |  |

Abbreviations: hemoglobin(Hb), platelets(Plt), white blood cell (WBC), red blood cell distribution Width(RDW), mean corpuscular hemoglobin concentration(MCHC), alanine aminotransferase(ALT), total bilirubin (TBIL), iserum creatinine(Scr), Sequential Organ Failure Assessment(SOFA), Geriatric Nutritional Risk Index (GNRI), Prognostic Nutritional Index (PNI), partial pressure of oxygen (PO₂), Red blood cell count (RBC), lactate (Lac), blood urea nitrogen (BUN), aspartate aminotransferase (AST).

Figure S2**:** Restricted cubic spline analysis based on logistic regression for the MIMIC database

Figure S3**:** Restricted cubic spline analysis based on logistic regression for the Xinhua Hospital database

Figure S4: Random Forest ranks the importance of factors contributing to SIC in MIMIC-IV database

Figure S5: Random Forest ranks the importance of factors contributing to SIC in Xinhua Hospital database

Figure S6: Correlation matrix between nutritional indicators and SIC components in MIMIC-IV database. The negatively relation is represented in red, and positive relation is represented in blue. The darker the colour, the higher the correlation is. *p < 0.05; **p < 0.01; ***p < 0.001.

Figure S7: Correlation matrix between nutritional indicators and SIC components in Xinhua Hospital database. The negatively relation is represented in red, and positive relation is represented in blue. The darker the colour, the higher the correlation is. *p < 0.05; **p < 0.01; ***p < 0.001.

Figure Abbreviations: hemoglobin(Hb), platelets(Plt), white blood cell (WBC), red blood cell distribution Width(RDW), mean corpuscular volume(MCV), albumin(Alb), aspartate aminotransferase (AST), total bilirubin (TB), international normalization ratio(INR), serum creatinine(Scr), lactate (Lac), Sequential Organ Failure Assessment(SOFA), Geriatric Nutritional Risk Index (GNRI), Prognostic Nutritional Index (PNI), sepsis-induced coagulopathy (SIC), heart rate(HR),Red blood cell count (RBC), blood urea nitrogen (BUN), mean corpuscular hemoglobin concentration(MCHC).
